# Epidemiologic Trends in Secondary Malignant Neoplasms of the Liver in the United States: A 25-Year National Study

**DOI:** 10.21203/rs.3.rs-7613109/v1

**Published:** 2025-09-16

**Authors:** Mahnoor Khan, Daniyal Ali Khan, Kantesh Kumar, Syed Ali Tayyeb Hasan, Shahzaib Khan, Fatima Najam, Munir Mehmood, Waqas Nawaz

**Affiliations:** Shaikh Khalifa Bin Zayed Al Nahyan Medical & Dental College; Aga Khan University; Aga Khan University; Aga Khan University; Rutgers Health Community Medical Center; Jefferson Abington Hospital; Aga Khan University; Ohio State University Wexner Medical Center

**Keywords:** Health Inequities, Epidemiology, Mortality, Liver Neoplasms, Neoplasm Metastasis

## Abstract

**Background:**

Secondary malignant neoplasms of the liver (SMNL) are more common than primary liver cancers but remain understudied. This study provides the first nationwide analysis of SMNL mortality trends and disparities in the US.

**Methods:**

This study analyzed SMNL-related (ICD-10: C78.7) CDC WONDER mortality data (1999–2023), and US Cancer Statistics incidence data for adults ≥ 45 years, stratified by demographics, geography, and primary site. Age-adjusted mortality and incidence rates (AAMRs, AAIRs) and crude mortality rates (CMRs) were reported per 100,000 population. Temporal trends were analyzed using Joinpoint regression.

**Results:**

SMNL-related AAMR rose from 23.8 (1999) to 25.1 (2023). Males accounted for 51.3% of the 597,332 deaths and had higher AAMRs than females throughout. Lung (25.6%) and colon (24.6%) cancers were the leading primary sites causing SMNL-related deaths. AAIRs and AAMRs (irrespective of metastasis) declined for most primary cancers. CMRs progressively increased across each successive 10-year age group. Non-Hispanic (NH) Blacks had the highest AAMR but with a declining trend, while NH Whites saw a significant increase. West’s AAMRs showed the biggest increase. Rural areas consistently had higher AAMRs.

**Conclusions:**

SMNL mortality is rising among older US adults, with disparities. Improved primary cancer survival extends the window for metastatic progression, highlighting the need for better detection and management.

## 1 Introduction

Secondary malignant neoplasm of the liver (SMNL) is often more prevalent than primary liver cancer, yet its mortality trends remain underexplored.^1^ This has been represented in a University of Cologne study where liver metastases (611 cases) outnumbered primary hepatocellular carcinoma (380 cases) among 1,357 tumor samples.^2^

Liver cancer is the sixth most common cancer worldwide, ranking fifth in men and ninth in women.^3^ In 2022, the US had the second-highest liver cancer incidence (43,492 cases) and the third-highest mortality (30,931 deaths).^3^ The liver is a common site for organ-specific metastasis due to its unique microvascular circulation.^4,5^ Gastrointestinal, breast, and prostate cancers, along with neuroendocrine tumors and sarcomas, commonly metastasize to the liver.^6^

While some studies have examined the burden of SMNL in the US using the Surveillance, Epidemiology, and End Results (SEER) database, they provide only limited coverage due to incomplete geographic representation and inconsistent registry inclusion over time.^6,7^ Although primary liver cancer mortality trends have been previously studied, no large-scale analysis has comprehensively examined SMNL mortality across demographic and geographic factors.^8^ Liver cancer, often diagnosed at advanced stages, has a poor prognosis due to its rapid progression and the liver’s vulnerability to metastasis, necessitating a nationwide analysis.^9^

This study addresses that gap by providing the first nationwide evaluation of SMNL mortality trends, highlighting demographic and geographic disparities and the primary cancers most commonly leading to SMNL-related mortality, offering insights for targeted surveillance and treatment strategies.

## 2 Materials and Methods

### 2.1 Study design and population

We conducted a population-based cross-sectional study primarily focused on SMNL-related mortality trends. Mortality data from 1999–2023 were obtained from the CDC’s Wide-Ranging Online Data for Epidemiologic Research (CDC WONDER), using the Multiple Cause of Death database.^10,11^ Individuals aged ≥ 45 years with SMNL were identified using the International Classification of Diseases, Tenth Revision (ICD-10) code C78.7.

To contextualize SMNL-related mortality, we examined both incidence and mortality trends for the primary cancers most associated with SMNL-related deaths. Incidence data from 1999–2021 were obtained from the United States Cancer Statistics (USCS) database, the official federal source for cancer surveillance. USCS is produced by the CDC and the National Cancer Institute (NCI), incorporating data from the National Program of Cancer Registries (NPCR) and the SEER program, covering nearly the entire US population.^12^

This study adhered to the Strengthening the Reporting of Observational Studies in Epidemiology (STROBE) guidelines and the Declaration of Helsinki.^13^ Since the study used publicly available, deidentified data, informed consent and institutional review board approval were not required.

### 2.2 Outcome Variables

This study primarily analyzed SMNL-related mortality in the US, including (1) trends in overall age-adjusted mortality rates (AAMR); (2) AAMR trends stratified by gender, race, region, urbanization status, primary cancer site, and the three states with the highest and lowest average annual percentage changes (AAPC); (3) trends in crude mortality rates (CMR) across different age groups; (4) trends in the proportion of deaths by place of death; and (5) state-level variation in overall AAMR and AAPC. The secondary objectives were to examine trends in AAIR and AAMR for the top ten primary cancers most frequently associated with SMNL-related mortality, using overall cancer-specific incidence and mortality data irrespective of SMNL contribution.

### 2.3 Data abstraction

Gender was used as a binary variable. Race/ethnicity classifications included non-Hispanic (NH) White, NH Black or African American (NH-BAA), NH Asian or Pacific Islander (NH-A/PI), NH American Indian or Alaska Native (NH-AI/AN), and Hispanic. Age groups were stratified into 45–54, 55–64, 65–74, 75–84, and ≥ 85 years. Places of death were classified as inpatient hospital (IPH), outpatient or emergency room facility (OEF), the decedent’s home, hospice facility, and nursing home/long-term care facility. According to the 2013 US Census classification, the population was categorized using the National Center for Health Statistics Urban-Rural Classification Scheme as urban (≥ 50,000) or rural (< 50,000).^14^ AAMR by urbanization status was available only for 1999–2020. Regions were classified into Northeast, Midwest, South, and West according to the Census Bureau definitions. Primary cancer sites were identified using concurrent ICD-10 codes with SMNL on death certificates **(Supplementary Table 1)**. Analyses for prostate and ovarian cancers were restricted to males and females, respectively. Breast cancer analysis focused on females, with a sensitivity analysis combining both genders. Male breast cancer was excluded due to suppressed or negligible AAMRs (< 0.1).

To protect confidentiality, CDC WONDER suppresses counts below 10 and flags rates as unreliable for counts under 20. Subgroups with small numbers in our study included such suppressed or unreliable data, indicated by “−” in tables and dotted lines in figures.

### 2.4 Statistical analysis

AAMRs, AAIRs, and CMRs per 100,000 individuals were calculated. The 2000 US population served as the standard for AAMRs and AAIRs.^15^ The Joinpoint Regression Program (version 5.3.0) was used to compute annual percent changes (APCs) and AAPC, as well as 95% confidence intervals (CI), using log-linear regression models. Temporal variations were recorded to identify significant shifts in AAMRs and AAIRs. The statistical significance of the APCs that were computed for each segment connecting join points was determined using the Monte Carlo permutation test. A two-tailed t-test (P < 0.05, indicated by an asterisk “*” in data and graphics) was used to assess changes in APC. A significant deviation of the mortality trend slope from zero classified the change as increasing or decreasing.

## 3 Results

**Supplementary Fig. 1** provides a central illustration of our study’s SMNL-related mortality summary. Detailed results of the study are shown in Figs. 1–4, **supplementary tables 1–14**, and **supplementary Figs. 2–11.**

### 3.1 Overall

**Figure 1.**
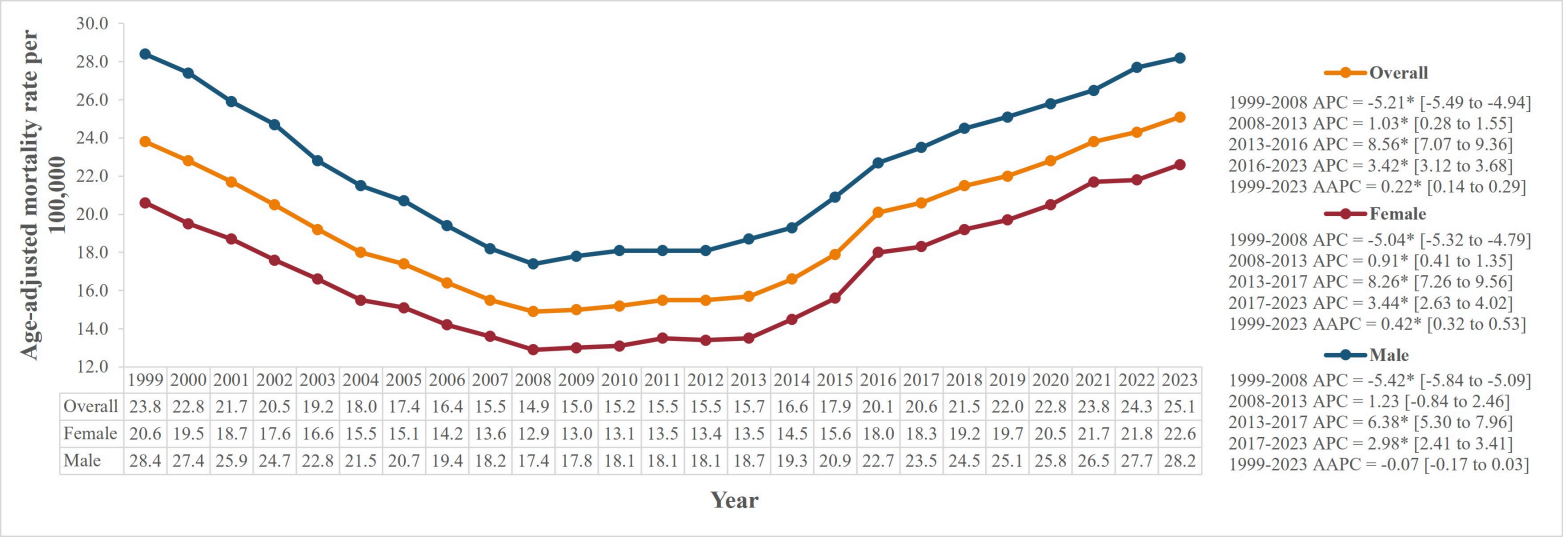
Age-adjusted mortality rates per 100,000 overall and stratified by gender Statistically significant values have been represented by an asterisk “*”.

Between 1999 and 2023, the study reported 597,332 SMNL-related deaths among adults aged 45 and above **(Supplementary Table 2)**. For overall SMNL-related mortality, AAMR increased from 23.8 per 100,000 in 1999 to a peak of 25.1 in 2023 (AAPC = 0.22%*, 95% CI: 0.14 to 0.29) (Fig. 1). The AAMR initially declined significantly from 23.8 in 1999 to 14.9 in 2008 (APC= −5.21%*, 95% CI: −5.49 to −4.94). This was followed by a significant rise, especially from 15.7 in 2013 to 20.1 in 2016 (APC = 8.56%*, 95% CI: 7.07 to 9.36). **(Supplementary Table 3)**.

### 3.2 Gender Stratified

Of the total SMNL-related deaths, 306,558 (51.3%) were men, with consistently higher AAMR than women from 1999 to 2023 (Fig. 1). Both genders followed similar mortality trends throughout, with a significant initial decline from 1999 till 2008. During this period, AAMR almost halved from 20.6 to 12.9 for females (APC=−5.04%*, 95% CI: −5.32 to −4.79) and 28.4 to 17.4 for men (APC=−5.42%*, 95% CI: −5.84 to −5.09). Following this period, AAMR kept increasing till 2023 for both genders, with AAMR of females reaching a peak of 22.6 in 2023 but that of males reached 28.2, close to their peak of 28.4 in 1999. The initial rise from 2008 to 2013 was significant only for females (females: APC = 0.91%*, 95% CI: 0.41 to 1.35; males: APC = 1.23%, 95% CI: −0.84 to 2.46). The steepest rise in AAMR was observed by both genders from 2013 to 2017. AAMR for females increased from 13.5 in 2013 to 18.3 in 2017 (APC = 8.26%*, 95% CI: 7.26 to 9.56), and from 18.7 to 23.5 for males (APC = 6.38%*, 95% CI: 5.30 to 7.96).

### 3.3 Primary cancer site stratified

**Figure 2.**
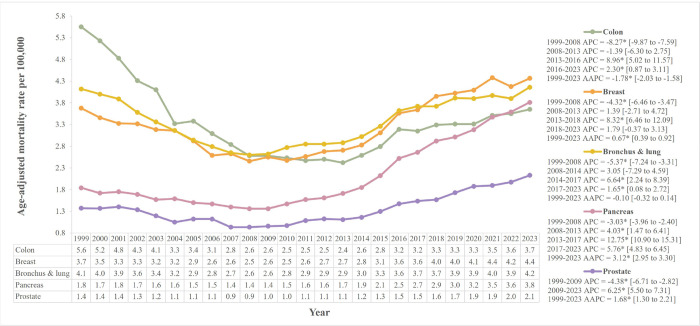
Age-adjusted mortality rates per 100,000 stratified by primary cancer sites that led to the highest SMNL-related mortality (with SMNL selected as a contributing cause of death) Statistically significant values have been represented by an asterisk “*”.

The top five primary cancers associated with the highest SMNL-related mortality were bronchus and lung, colon, breast, pancreas, and prostate (Fig. 2). Bronchus and lung cancer accounted for the most SMNL-related deaths (104,893), while colon cancer, though second in deaths (100,846), had the highest initial AAMR. All the top five cancers shared an initial significant drop in AAMR till 2008 followed by a rising trend till 2023. Of these, only colon cancer showed an overall significant decline in AAMR, dropping from 5.6 in 1999 to 3.7 in 2023 (AAPC=−1.78%*, 95% CI: − 2.03 to − 1.58). Its most significant drop in AAMR was from 1999 to 2008, reaching 2.6 (APC=−8.27%*, 95% CI: −9.87 to −7.59). Although, it kept a significantly increasing trend after 2013, especially from 2.4 in 2013 to 3.2 in 2016 (APC = 8.96%*, 95% CI: 5.02 to 11.57), it could only reach almost two-thirds of its value in 1999. Bronchus and lung became the leading cause of SMNL-related death in 2010, with an insignificant overall change in AAMR from 4.1 in 1999 to 4.2 in 2023 (AAPC=−0.10%, 95% CI: 0.32 to 0.14). Female breast cancer’s AAMR declined from 3.7 in 1999 to 2.5 in 2008 (APC=−4.32%*, 95% CI: −6.46 to −3.47), then steadily rose, becoming the leading cause of SMNL-related mortality by 2018. Initially third in 1999, its AAMR reached 4.4 in 2023 (AAPC = 0.67%*, 95% CI: 0.39 to 0.92).

Pancreatic cancer showed the largest overall rise in AAMR, nearly doubling from 1.8 in 1999 to 3.8 in 2023 (AAPC = 3.12%*, 95% CI: 2.95 to 3.30). It surpassed colon cancer to become the third leading cause of SMNL-related mortality in 2023. Prostate cancer’s AAMR rose by 1.5 times from 1.4 in 1999 to 2.1 in 2023 (AAPC = 1.68%*, 95% CI: 1.30 to 2.21). Other primary cancers contributing to SMNL-related mortality, such as esophageal, stomach, rectal, ovarian, and renal cancers, have been presented in **Supplementary Tables 4 and 5**.

Between 1999 and 2021, AAIR declined for colon, bronchus and lung, and prostate cancers, while breast and pancreatic cancers showed relative stability or modest increases **(Supplementary Fig. 2, Supplementary Tables 6–8)**. Colon cancer incidence declined steadily from 113.3 to 65.0, nearly halving over the period (APC=−2.79%*, 95% CI: − 2.98 to − 2.60). Prostate cancer, despite initial fluctuations, showed the sharpest overall decline from 483.5 to 327.8 (AAPC=−1.77%*, 95% CI: − 2.20 to − 1.19), with a significant drop between 2008–2014 (APC=−6.29%*, 95% CI: − 12.6 to − 3.97). Bronchus and lung cancer incidence also decreased significantly (AAPC=−1.70%*, 95% CI: − 1.92 to − 1.46), particularly between 2006–2019. Only pancreatic cancer observed a significant overall rise in AAIR from 30.3 to 36.9 (AAPC = 0.85%*, 95% CI: 0.78 to 0.93). Female breast cancer incidence declined from 336.3 to 298.4 by 2005, but subsequently increased, resulting in a non-significant overall AAPC of − 0.27% (95% CI: − 0.48 to 0.05). Supplementary analysis of breast cancer in both genders showed AAMRs and AAIRs nearly half those of female breast cancer **(Supplementary Figs. 3 and 4)**.

Trends in AAMRs for the top ten primary cancers leading to SMNL-related mortality, excluding SMNL as a contributing cause, showed a significant decline for all cancers except pancreatic cancer. Pancreatic cancer experienced a modest increase in AAMR (APC = 0.35%*, 95% CI: 0.29–0.41) **(Supplementary Fig. 5, Supplementary Tables 9 and 10)**. AAMR for stomach, colon, bronchus and lung, ovarian, and prostate cancers nearly halved from 1999–2023.

### 3.4 Age Stratified

CMRs increased progressively across each successive 10-year age group, with a nearly 10-fold difference between ages 45–54 and 85+ **(Supplementary Fig. 6)**. Rates declined across all age groups in the early 2000s, plateaued around 2008, and remained stable until 2013. The 45–54 age group exhibited the highest AAPC (0.69%*, 95% CI: 0.48–0.89), with CMR rising from 6.1 to 7.3. At the other end, the 85 + group reached a peak of 70.7 in 2023 from 62.5 in 1999 (AAPC = 0.47%*, 95% CI: 0.21 to 0.73). The steepest rise occurred in the 55–64 group between 2013–2016 (APC = 11.46%*, 95% CI: 8.56–13.07), from 11.9 to 16.2. Only the 65–74 group showed a significant overall decline in CMR, from 37.6 to 36.5 (AAPC=−0.12%*, 95% CI: −0.20 to −0.04).

### 3.5 Race stratified

**Figure 3.**
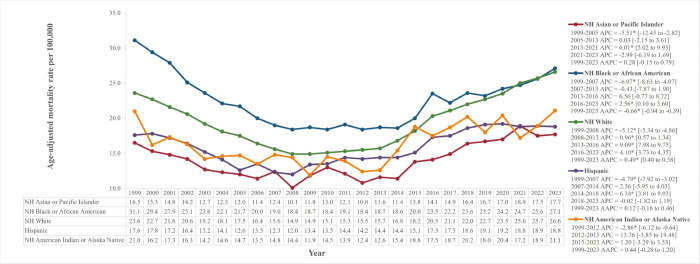
Age-adjusted mortality rates per 100,000 stratified by race Statistically significant values have been represented by an asterisk “*”.

Throughout the study period, NH-BAA had the highest AAMR, declining from 31.1 to 27.1 (AAPC=−0.66%*, 95% CI: −0.94 to −0.39) (Fig. 3). Initially, three-fourths of NH-BAAs’ AAMR, NH Whites saw a significant increase from 23.6 in 1999 to 26.6 in 2023 (AAPC = 0.49%*, 95% CI: 0.40 to 0.58), nearing NH-BAAs’ rates. Both groups experienced a nearly 40% decline in AAMR until 2008, after which mortality rose again. Although NH-A/PI, NH-AI/AN, and Hispanics initially saw significant declines in mortality, subsequent increases rendered their overall changes insignificant (NH-A/PI AAPC = 0.28%; NH-AI/AN AAPC = 0.44%; Hispanics AAPC = 0.12%).

### 3.6 Place of death stratified

50.0% of deaths occurred at home, followed by IPH (25.4%), nursing homes (12.2%), hospice facilities (10.9%), and OEF (1.5%) **(Supplementary Table 11)**. From 1999–2023, IPH deaths more than halved, declining from 38.9% to 19.0%, while home deaths increased from 43.9% to 56.2% **(Supplementary Fig. 7)**. Hospice deaths increased 31-fold (0.5% in 2003 to 15.5% in 2023), whereas nursing home deaths declined by nearly half (15.4% to 8.2%).

### 3.7 State stratified

**Figure 4.**
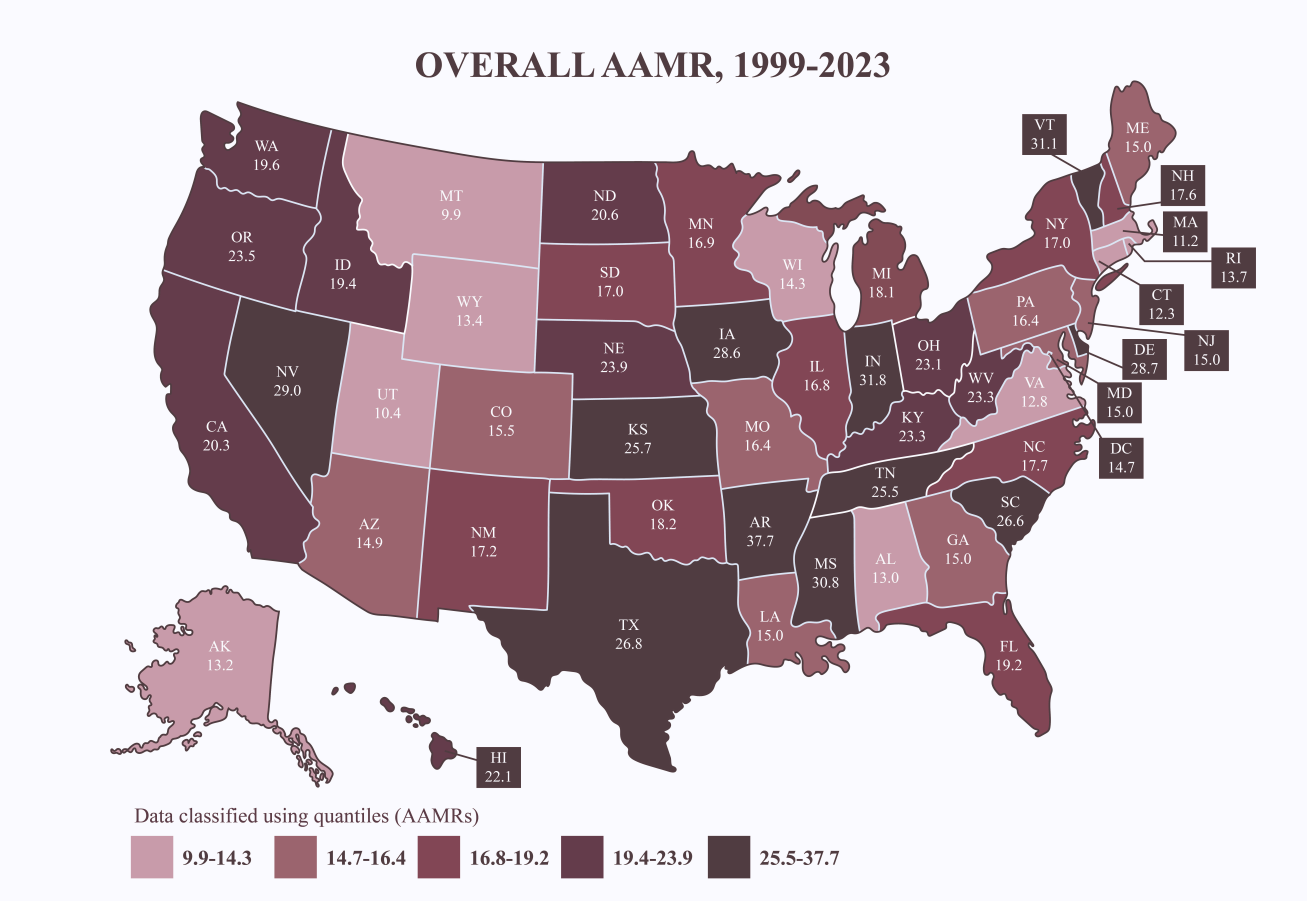
Overall age-adjusted mortality rates per 100,000 from 1999–2023 stratified by states

States in the upper 90th percentile for overall SMNL-related AAMR (≥ 28.7) included Arkansas (37.7), Indiana (31.8), Vermont (31.1), Mississippi (30.8), Nevada (29.0), and Delaware (28.7) (Fig. 4). In contrast, states in the 10th percentile (≤ 13.0) were Alabama (13.0), Virginia (12.8), Connecticut (12.3), Massachusetts (11.2), Utah (10.4), and Montana (9.9). AAMR rose the most in Vermont (AAPC = 5.30%*, 95% CI: 3.40–8.09), Oregon (AAPC = 4.90%*, 95% CI: 4.33–5.56), and Hawaii (AAPC = 2.62%*, 95% CI: 1.77–3.54) **(Supplementary Figs. 8 and 9; Supplementary Tables 12–14)**. The largest declines were seen in the District of Columbia (DC) (AAPC=−5.01%*, 95% CI: −6.55 to 3.25), New Jersey (AAPC=−4.09%*, 95% CI: −4.50 to −3.69), and Rhode Island (−2.92%*, 95% CI: −3.75 to −2.13).

#### 3.8 Region stratified

While all US census regions showed an initial decline in SMNL-related mortality, only the Northeast had an overall decrease in AAMR (AAPC=−1.83%*, 95% CI: −2.05 to −1.61), dropping from 24.9 in 1999 to 16.3 in 2023 **(Supplementary Fig. 10)**. By 2023, the Northeast’s AAMR was approximately two-thirds that of the other regions. In contrast, the West, which began with the lowest AAMR (19.8), saw the largest increase to 25.8 (AAPC = 1.24%*, 95% CI: 0.97 to 1.51). The South also experienced a significant rise (AAPC = 0.47%*, 95% CI: 0.29 to 0.66), while the Midwest showed a non-significant increase (AAPC = 0.17%, 95% CI: −0.02 to 0.34).

#### 3.9 Urbanization status stratified

Rural areas consistently had higher mortality rates, with no significant overall change (AAPC = 0.16%, 95% CI: −0.08 to 0.38) **(Supplementary Fig. 11)**. In contrast, urban areas saw a significant decline from 23.5 in 1999 to 21.9 in 2020 (AAPC=−0.24%*, 95% CI: −0.36 to −0.11). After an initial drop in both settings, AAMRs began to rise, with the steepest increase occurring between 2013 and 2016 (Rural APC = 9.55%*, 95% CI: 6.41 to 11.62; Urban APC = 7.99%*, 95% CI: 6.43 to 8.97).

## 4 Discussion

### 4.1 Main findings of this study

Our study provides a comprehensive 25-year overview (1999–2023) of SMNL-related AAMRs in US adults aged ≥ 45. While AAMRs declined until 2008, a reversal occurred thereafter, with a particularly sharp increase between 2013 and 2016. This upward trend raises critical questions about the interplay between survivorship, late effects of cancer, and the healthcare system response. Notably, this increase occurred despite a significant decline in both AAIRs and AAMRs for most primary cancers, irrespective of metastasis, that were most frequently linked to SMNL-related mortality in our study. These trends likely reflect improved survival among patients with primary cancers, resulting in a longer window for metastatic progression, especially to the liver. Our study also identified notable disparities with higher mortality rates among males, NH-BAA individuals, older age groups, and those in rural areas. These disparities necessitate focused public health initiatives.

### 4.2 What is already known on this topic

Improved survivorship means that as patients live longer following a primary cancer diagnosis, their risk of developing late metastatic disease correspondingly rises. This is supported by prior studies showing high rates of metastasis among cancer survivors. Out of 150,000 metastatic breast cancer patients, 75% were initially diagnosed with Stage II/III cancer.^16^ Moreover, as noted by Mani et al, metastatic disease remains the principal cause of cancer-related mortality.^17^ In this context, our findings underscore both the successes and emerging challenges of modern cancer care.

Prior literature shows that males face a higher burden of cancers prone to liver metastasis, such as prostate cancer, and are twice as likely as females to present with liver metastases.^18^ Similarly, NH-BAA individuals are more often diagnosed at later stages. Akinyemiju et al found higher rates of de novo metastasis in NH-BAA with breast, colorectal, and prostate cancer compared to NH Whites (11% vs 9%).^19^

### 4.3 What this study adds

To our knowledge, this is the first study to analyze SMNL-related mortality trends in the US, with important policy implications. Although the rise in AAMRs after 2008 may appear alarming, it likely reflects advances in cancer treatment, screening, and awareness, which have prolonged survival and, in turn, increased opportunities for late metastatic disease. These findings highlight the need for long-term survivorship strategies, including routine monitoring for metastatic spread in high-risk groups.

The gender- and race-based disparities observed underscore the need for equity-focused cancer control policies. Males had consistently higher AAMRs, which may be attributable to the higher incidence of male-dominant cancers, such as prostate cancer, that often metastasize to the liver and carry poorer prognoses. The higher AAMRs among NH-BAA likely reflect a combination of delayed cancer diagnosis, more advanced disease at presentation, and systemic inequities in access to timely and effective cancer care. NH Whites were the only group to show a significant increase in AAMRs over time, reaching rates nearly equal to those of NH-BAA. This may reflect longer survival after primary cancer diagnosis, providing more time for metastasis, a pattern also observed in the higher CMRs among older adults in our study.^20–22^ These racial disparities likely stem from a mix of socioeconomic, access-related, and systemic factors.^23^ Although our study did not directly assess these drivers, it highlights persistent inequities in cancer outcomes across the US healthcare system.

Targeted resource allocation is essential to improve early detection, timely diagnosis, and access to effective treatment in underserved communities. Integrating stratified risk assessments into survivorship protocols, based on demographic and clinical profiles, may help optimize monitoring and intervention strategies.

### 4.4 Limitations of this study

Our study utilized the CDC WONDER database; as a result, it is subject to misclassification or incomplete reporting, as it is dependent on death certificate documentation. In rare cases with multiple malignancies, it was not possible to determine the primary source of SMNL, as it was based on co-listed ICD-10 codes on death certificates. The cross-sectional design limits causal inference. Urban-rural analysis was restricted to data up to 2020 due to unavailable population estimates and AAMRs thereafter. Additionally, subgroups with fewer than 10 deaths were suppressed, and those with fewer than 20 were considered statistically unreliable, including data from hospice facilities and select states.

## 5 Conclusion

Despite declining mortality from most primary cancers, AAMRs for SMNL have steadily increased in the US since 2008. This pattern likely reflects that the advancements in the early detection and treatment of primary tumor sources have resulted in increased survival, leading to an increased chance of metastatic progression, particularly to the liver. Our study also highlighted several disparities in SMNL-related mortalities by gender, age, and race, with males, older adults, and NH-BAA having the highest mortality rates. These findings support the need for enhanced long-term surveillance after the initial treatment of primary tumors, for the early diagnosis of recurrence, and to prevent metastasis.

## Supplementary Material

Supplementary Files

This is a list of supplementary files associated with this preprint. Click to download.

• SupplementaryAppendixTablesandFiguresfinal.docx

## Data Availability

Data were abstracted from the Centers for Disease Control and Prevention’s Wide-Ranging Online Data for Epidemiologic Research (CDC-WONDER) platform. All relevant data are included in the manuscript and supplementary materials. CDC-WONDER Multiple Cause of Death 1999–2020: https://wonder.cdc.gov/mcd-icd10.html CDC-WONDER Multiple Cause of Death 2018–2023: https://wonder.cdc.gov/mcd-icd10-expanded.html

## References

[1] Ananthakrishnan A, Gogineni V, Saeian K. Epidemiology of primary and secondary liver cancers. Semin Intervent Radiol 2006; 23(1): 47–63. 21326720 10.1055/s-2006-939841 PMC3036307

[2] Kasper HU, Drebber U, Dries V, Dienes HP. [Liver metastases: incidence and histogenesis]. Z Gastroenterol 2005; 43(10): 1149–57. 16220456 10.1055/s-2005-858576

[3] Fund WCR. Liver cancer statistics. https://www.wcrf.org/preventing-cancer/cancer-statistics/liver-cancer-statistics/ (accessed Feb 11 2025).

[4] Nguyen DX, Bos PD, Massagué J. Metastasis: from dissemination to organ-specific colonization. Nat Rev Cancer 2009; 9(4): 274–84. 19308067 10.1038/nrc2622

[5] Jin K, Gao W, Lu Y, Lan H, Teng L, Cao F. Mechanisms regulating colorectal cancer cell metastasis into liver (Review). Oncol Lett 2012; 3(1): 11–5. 22740847 10.3892/ol.2011.432 PMC3362390

[6] Horn SR, Stoltzfus KC, Lehrer EJ, Epidemiology of liver metastases. Cancer Epidemiol 2020; 67: 101760. 32562887 10.1016/j.canep.2020.101760

[7] Wang ZG, He ZY, Chen YY, Gao H, Du XL. Incidence and survival outcomes of secondary liver cancer: a Surveillance Epidemiology and End Results database analysis. Transl Cancer Res 2021; 10(3): 1273–83. 35116454 10.21037/tcr-20-3319 PMC8797763

[8] Altekruse SF, Henley SJ, Cucinelli JE, McGlynn KA. Changing hepatocellular carcinoma incidence and liver cancer mortality rates in the United States. Am J Gastroenterol 2014; 109(4): 542–53. 24513805 10.1038/ajg.2014.11 PMC4148914

[9] Liu JH, Chen PW, Asch SM, Busuttil RW, Ko CY. Surgery for hepatocellular carcinoma: does it improve survival? Ann Surg Oncol 2004; 11(3): 298–303. 14993025 10.1245/aso.2004.03.042

[10] [dataset]* Centers for Disease Control and Prevention, National Center for Health Statistics. National Vital Statistics System, Mortality 1999–2020 on CDC WONDER Online Database, released in 2021. Data are from the Multiple Cause of Death Files, 1999–2020, as compiled from data provided by the 57 vital statistics jurisdictions through the Vital Statistics Cooperative Program. https://wonder.cdc.gov/mcd-icd10.html (accessed April 7 2025).

[11] [dataset]* Centers for Disease Control and Prevention, National Center for Health Statistics. National Vital Statistics System, Mortality 2018–2023 on CDC WONDER Online Database, released in 2024. Data are from the Multiple Cause of Death Files, 2018–2023, as compiled from data provided by the 57 vital statistics jurisdictions through the Vital Statistics Cooperative Program. http://wonder.cdc.gov/mcd-icd10-expanded.html (accessed April 7 2025).

[12] [dataset]* United States Cancer Statistics - Incidence: 1999 – 2021, WONDER Online Database. United States Department of Health and Human Services, Centers for Disease Control and Prevention and National Cancer Institute; 2023 submission; 2024 release. http://wonder.cdc.gov/cancer-v2021.html (accessed April 8 2025).

[13] Cheng A, Kessler D, Mackinnon R, Reporting Guidelines for Health Care Simulation Research: Extensions to the CONSORT and STROBE Statements. Simul Healthc 2016; 11(4): 238–48. 27465839 10.1097/SIH.0000000000000150

[14] Ingram DD, Franco SJ. 2013 NCHS Urban-Rural Classification Scheme for Counties. Vital Health Stat 2 2014; (166): 1–73. 24776070

[15] Anderson RN, Rosenberg HM. Age standardization of death rates: implementation of the year 2000 standard. Natl Vital Stat Rep 1998; 47(3): 1–16, 20. 9796247

[16] American Cancer Society. Breast Cancer Facts & Figures 2022–2024. Atlanta: American Cancer Society, Inc. 2022. https://www.cancer.org/content/dam/cancer-org/research/cancer-facts-and-statistics/breast-cancer-facts-and-figures/2022-2024-breast-cancer-fact-figures-acs.pdf#:~:text=More%20than%204%20million%20US,III%20cancer.24 (accessed June 10 2025).

[17] Mani K, Deng D, Lin C, Wang M, Hsu ML, Zaorsky NG. Causes of death among people living with metastatic cancer. Nature Communications 2024; 15(1): 1519. 10.1038/s41467-024-45307-x PMC10876661 38374318

[18] Buxton AK, Abbasova S, Bevan CL, Leach DA. Liver Microenvironment Response to Prostate Cancer Metastasis and Hormonal Therapy. Cancers 2022; 14(24): 6189. 36551674 10.3390/cancers14246189 PMC9777323

[19] Akinyemiju T, Sakhuja S, Waterbor J, Pisu M, Altekruse SF. Racial/ethnic disparities in de novo metastases sites and survival outcomes for patients with primary breast, colorectal, and prostate cancer. Cancer Med 2018; 7(4): 1183–93. 29479835 10.1002/cam4.1322 PMC5911612

[20] Aizer AA, Wilhite TJ, Chen MH, Lack of reduction in racial disparities in cancer-specific mortality over a 20-year period. Cancer 2014; 120(10): 1532–9. 24863392 10.1002/cncr.28617

[21] Stenzel AE, Buas MF, Moysich KB. Survival disparities among racial/ethnic groups of women with ovarian cancer: An update on data from the Surveillance, Epidemiology and End Results (SEER) registry. Cancer Epidemiol 2019; 62: 101580. 31400533 10.1016/j.canep.2019.101580

[22] Kucera CW, Tian C, Tarney CM, Factors Associated With Survival Disparities Between Non-Hispanic Black and White Patients With Uterine Cancer. JAMA Netw Open 2023; 6(4): e238437. 37067801 10.1001/jamanetworkopen.2023.8437 PMC10111180

[23] Haider AH, Scott VK, Rehman KA, Racial disparities in surgical care and outcomes in the United States: a comprehensive review of patient, provider, and systemic factors. J Am Coll Surg 2013; 216(3): 482–92.e12. 23318117 10.1016/j.jamcollsurg.2012.11.014 PMC5995336

